# Optimization of a Perovskite Oxide-Based Cathode Catalyst Layer on Performance of Direct Ammonia Fuel Cells

**DOI:** 10.1021/acsami.2c17253

**Published:** 2022-12-27

**Authors:** Georgina Jeerh, Peimiao Zou, Mengfei Zhang, Shanwen Tao

**Affiliations:** † School of Engineering, University of Warwick, CoventryCV4 7AL, U.K.; ‡ Department of Chemical Engineering, Monash University, Clayton, Victoria3800, Australia

**Keywords:** direct ammonia fuel cell (DAFC), perovskite, cathode, catalyst layer, optimization

## Abstract

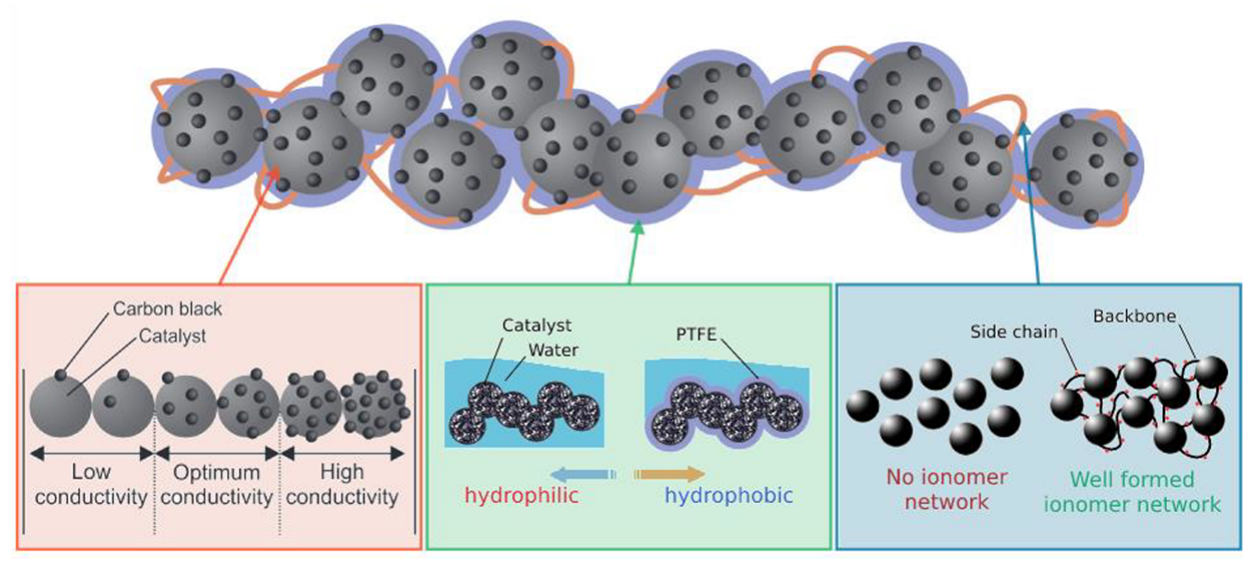

To maximize fuel cell performance, transport pathways for electrons, ions, and reactants should be connected well. This demands a well-constructed microstructure in the catalyst layer (CL). Herein we design and optimize a cathode CL for a direct ammonia fuel cell (DAFC) using a perovskite oxide as the catalyst to reduce reliance on platinum group metals (PGMs). The effects of tailoring carbon, ionomer, and polytetrafluoroethylene (PTFE) content in cathode CLs (CCLs) were explored, and several DAFCs were tested. Using the same catalyst and operating conditions, the lowest maximum current density and peak power density obtained were 85.3 mA cm^–2^ and 5.92 mW cm^–2^, respectively, which substantially increased to 317 mA cm^–2^ and 30.1 mW cm^–2^ through proper carbon, ionomer, and PTFE optimization, illustrating the importance of an effective three-phase interface. The findings reveal that despite employment of an active catalyst for oxygen reduction at the cathode site, the true performance of the catalyst cannot be reflected unless it is supported by proper design of the CCL. The study also reveals that by optimizing the CCL, similar performances to those of Pt/C-based CCLs in literature can be obtained at a cost reduction.

## 1 Introduction

Fuel cells have received considerable attention as power devices in sectors ranging from automotive power to microelectronics due to their efficiency, simplicity, fast start-up, and low operating temperature. Although fuel cell technology stands on the brink of large-scale commercialization, it is greatly limited by factors such as durability and cost. The automotive industry, for example, is especially demanding and requires sufficient cost reductions to be made for fuel cells to be cost-competitive with current technologies in the sector.^1^

To maximize membrane electrode assembly (MEA) performance, the transport pathways of electrons, ions, and reactants should be connected well. This demands a well-constructed microstructure in the catalyst layer (CL).^2,3^ The development of high performance and robust CLs can play a key role in reducing cost, increasing power density, and prolonging cell life.^2^

A successful CL not only must be able to catalyze the given reaction but also must demonstrate good (i) electron conductivity, (ii) ionic conductivity, and (iii) transport pathways for reactant/products to and from the active sites. To satisfy these requirements, carbon supports, ionomer materials, and void regions are often introduced to provide the electron and ionic conduction networks respectively and mass transport channels for the reactants and products. It is at these well-connected interfaces that the electrochemical reactions take place; therefore design of such interfaces is of utmost importance for achieving high utilization of the catalyst material and improving cell performance.^4−7^

Although the optimization of CLs on the performance and durability of conventional proton exchange membrane fuel cells (PEMFCs) has been widely documented, these cells remain hindered due to their heavy reliance on group metals (PGMs) such as Pt/C.^8^ This has prompted search for alternative types of fuel cells.

Research toward alkaline exchange membrane fuel cells (AEMFCs) has been prompted by the possibility of utilizing lower costing and more sustainable non-PGMs due to the absence of harsh, acidic conditions.^8^ Direct ammonia fuel cells (DAFCs) are a particularly interesting branch of alkaline exchange membrane fuel cells (AEMFCs) that not only alleviate dependency on PGMs but also make use of ammonia’s high energy density, large-scale global production, extensive existing infrastructure, and low cost per unit energy.^8−19^ This makes them particularly attractive alternatives to PEMFCs. Regardless of such advantages, optimization of CLs in these cells is not widely explored, despite the clear benefits of doing so in PEMFCs.

Even though CCL optimization has not been explored in DAFCs, the fundamental requirements of a successful CL can be adopted from PEMFCs, that being a CL that not only catalyzes the given reaction but also provides transport networks for electronic, ionic, and reactant pathways. Perovskite oxides offer excellent catalytic activity toward oxygen reduction reaction (ORR) under alkaline conditions and therefore serve as superb cathode catalysts in DAFCs but do not incorporate carbon into their conventional sol–gel synthesis method and the effect of mixing different perovskite catalyst to carbon ratios on CCL performance in DAFCs has not been explored.^20^ This opens doors for further optimization of CCLs in AEMFCs.

Some perovskite oxides have inherently low electrical conductivity at low temperature; therefore exploiting the true electrocatalytic activity of these catalysts is difficult.^20,21^ To overcome this limitation, addition of carbon to form perovskite/carbon composites is a useful strategy to improve ORR activity and provide good electronic conductivity.^22,23^ Addition of CB has therefore become a common method to measure and compare ORR activity of transition metal oxides.^20,22,23^ These studies highlight the importance of properly tailoring perovskite to carbon ratios on ORR performance; yet these ratios may not directly relate to the best ratio in CCLs and such an effect has not widely been studied in the adoption of CCLs. In a previous paper, we were first to mix perovskite oxide to CB in an arbitrary ratio of 5:1 to improve electrical conductivity of the non-PGM-based cathode for use in a DAFC.^22,23^ We also more recently explored a fixed perovskite:carbon ratio in a study regarding perovskite oxides as efficient cathode catalysts in DAFCs.^24^ However, neither study explores the effects of properly tailoring and optimizing the perovskite:carbon ratio on performance of the cell to truly appreciate the impact of a good electrical conductivity network throughout the CCL.

The effects of hydrophobicity in CLs have extensively been explored in PEMFCs. These devices must effectively manage water generation at the cathode since the presence of water can fill pores in the gas diffusion layer and hinder gas transport leading to reactant starvation. Distinctive pathways for gas and liquid transport should therefore be provided to reduce reactant starvation. Hydrophobic nanoparticles such as PTFE or Nafion are typically added to the CL structure to create two-phase flow for excess water removal and easy access for reactants to active catalyst sites.^25^ Although the specific effects of varying hydrophobicity in CCLs of DAFCs have not been as widely investigated, it has been noted that the presence of a hydrophobic agent (PTFE) in the catalyst layer can significantly improve DAFC performance and durability. Wang et al. showed that the rate of water exiting the cathode exhaust when the anode feed was aqueous ammonia was substantially higher than that measured when the anode feed was a hydrogen/water vapor mixture.^26^ The results revealed a significant rate of liquid water crossover from anode to cathode in DAFCs, with most water crossing through the cathode CL before exiting the exhaust. To reduce flooding tendency, a (GDE)-based cathode with PTFE was fabricated and an increase in the contact angle of water on the surface was found, thereby providing better resistance to water flooding and facilitation of oxygen transport in the CL. Compared to the catalyst coated membrane (CCM)-based cathode with no PTFE, the GDE-based cathode with PTFE displayed higher performance, validating the need of PTFE. However, the effects of varying hydrophobicity in CCLs to find optimum DAFC performance were not explored.

The role of ionomer networks in CL ink is also important for successful cell performance and has been a major focus in the past decade.^1,4,27,28^ Since ionic transportation is essential for fuel cell mechanisms, lack of an ionomer leads to increased ionic resistance, rendering it difficult to utilize parts of the catalyst interface as active sites.^29^ Nafion is a well-documented ionomer that is extensively used in PEMFCs to increase proton conductivity within CLs.^4^ Exploration into optimizing Nafion content and its contributing role in CLs is therefore widely known. Nafion, however, is not well suited for DAFCs due its high proton conductivity and inability to assist in hydroxide transportation. A suitable hydroxide-ion conducting agent must therefore be added to CLs of DAFCs. The ionomer in such systems must also be physically compatible with the membrane employed and possess electronic conductivity and high gas/water permeability.^1^ Recently, a PiperION BP-100 ionomer has been documented to exhibit excellent hydroxide conductivity potential and has shown superb performance in previous studies regarding DAFCs.^26,30,31^ Yet literature regarding the effect of varying ionomer ratio remains scarce.

Given the importance of a well-connected microstructure on cell performance, this work focuses on CCL design to truly exploit a perovskite oxide-based catalyst for DAFCs. In a previous study, it was found that LaCr_0.25_Fe_0.25_Co_0.5_O_3−δ_ (LCFCO) perovskite demonstrated superb intrinsic activity toward ORR; however proper CCL design was not comprehensively explored previously and is the focus of this study.^24^ Therefore, in this study the ratio of LCFCO to carbon black (Vulcan XC-72R), hydrophobic agent (PTFE), and ionomer (PiperION BP-100) was characterized and investigated toward their influence on DAFC performance. LaCr_0.25_Fe_0.25_Co_0.5_O_3-δ_ (LCFCO) perovskite was employed as the active electrocatalyst material due to its superb intrinsic activity toward ORR as demonstrated in a previous study.^24^ The influence, however, of proper CCL design was not comprehensively explored previously and is the focus of this study. The findings reveal that despite employment of an active catalyst for oxygen reduction at the cathode site, the true performance of the catalyst cannot be reflected unless it is supported by proper optimization of the CCL.

## 2 Experimental Section

### 2.1 Materials

La(NO_3_)_3_·6H_2_O (98+%, Fisher Scientific), Co(NO_3_)_2_·6H_2_O (99%, Fisher Scientific), Cr(NO_3_)_2_·6H_2_O (99%, Fisher Scientific), and Fe(NO_3_)_3_·9H_2_O (99+%, Fisher Scientific) were used as metal precursors with no further purification. Citric acid (99+%, Sigma-Aldrich) and ethylene glycol (Alfa Aesar) were used during the synthesis process. Carbon black (Vulcan XC-72R) was used as a highly conductive material. Pt/C was commercially purchased (commercial 20 wt % platinum on carbon black, Fuel Cell Store). Carbon cloth (Fuel Cell Store) was used as the diffusion substrate layer for fuel cell tests along with a 20 μm PiperION-A20-HCO3 TP-85 membrane (Versogen) and PiperION BP-100 ionomer (Versogen). Polytetrafluoroethylene (PTFE) was purchased as a suspension from Sigma-Aldrich and used as the hydrophobic agent. Other chemicals such as isopropanol and KOH were all analytical grade reagents purchased from Alfa Aesar.

### 2.2 Synthesis of Perovskite Powders

The perovskite powders were synthesized using a method described in a previous paper.^24^ In brief, appropriate amounts of the metal nitrate precursors were dissolved in an aqueous solution at room temperature. Citric acid was added in a molar ratio of 1:1.2:1.2 of total metal ions:citric acid:ethylene glycol. The resulting solution was stirred and heated to 120 °C. After the evaporation of water, the sample was heated to 410 °C to form an ash which was finely ground using an agate mortar and pestle. The powder was calcinated in air at 500 °C for 2 h with a heating/cooling rate of 5 ^o^C min^–1^ before being reground and further calcinated at 700 °C for 4 h with a heating/cooling rate of 3 ^o^C min^–1^ to obtain the final perovskite phase. The collected LaCr_0.25_Fe_0.25_Co_0.5_O_3-δ_ sample was labeled LCFCO.

### 2.3 Electrode Preparation

**Figure 1:**
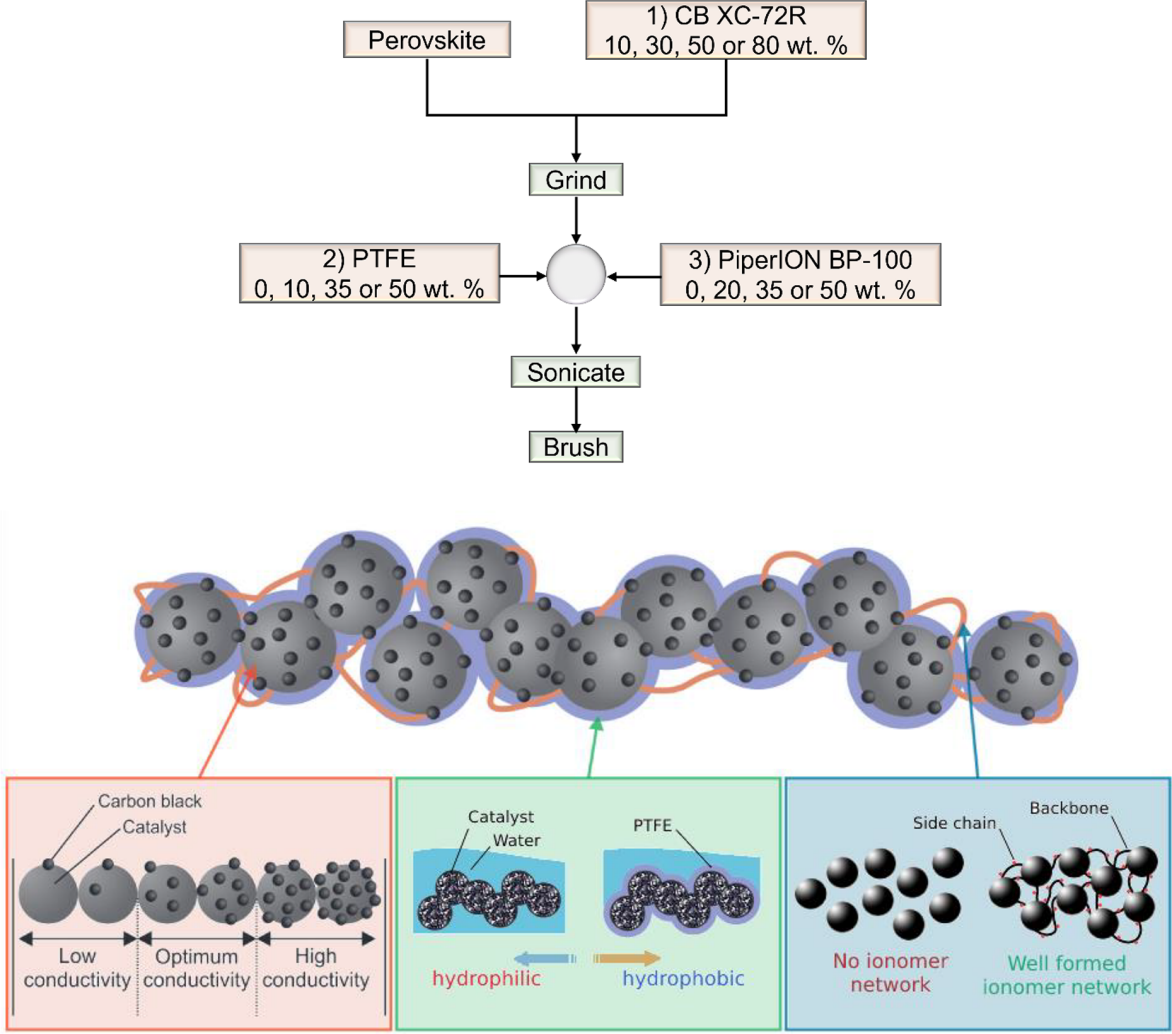
(a, top) General experimental procedure of electrode preparation with weight percentages given with respect to perovskite weight. (b, bottom) Illustration demonstrating the contributing effects of different components within CCLs for DAFCs.

Carbon cloth was washed and sonicated in dilute hydrochloric acid, propanol, and deionized water. The effects of carbon black (CB), ionomer, and PTFE content on fuel cell performance were sequentially tested, and the general procedure for making the CCL is shown in Figure 1. First, the perovskite oxide powder was physically mixed with high surface area CB (Vulcan XC-72R) at 10, 30, 50, or 80 wt % relative to the perovskite weight to find the optimum carbon content, hereby referred to as CCL-C10, CCL-C30, CCL-C50, and CCL-C80, respectively. Utilizing propanol as the solvent, the PiperION BP-100 ionomer was ultrasonicated in an ice–water bath for 1 h with perovskite/CB using an arbitrary amount of PTFE and PiperION BP-100 at 10 and 20 wt % relative to the perovskite weight, respectively. The ink was then brushed onto the carbon cloth and left to dry. Loading of oxide in the CCL-C10, CCL-C30, CCL-C50, and CCL-C80 was found to be 1.28, 1.24, 1.23, and 1.32 mg_oxide_ cm^–2^, respectively. The CB content delivering optimum performance was then used as a baseline as PTFE content was explored. Three further CCLs were prepared with PTFE content varied to 0, 35, or 50 wt %, hereby referred to as CCL-P0, CCL-P35, and CCL-P50, respectively. CCL-C50 (10 wt % PTFE) was also compared in this series. The loading of oxide in CCL-P0, CCL-P35, and CCL-P50 was found to be 1.20, 1.21, and 1.28 mg_oxide_ cm^–2^, respectively. Upon optimization of both CB and PTFE, PiperION BP-100 ionomer content was investigated. Three final CCLs were assembled, where ionomer was varied to 0, 35, or 50 wt %, hereby referred to as CCL-I0, CCL-I35, and CCL-I50, respectively. Again, for comparative purposes, CCL-C50 (20 wt % ionomer) was also tested. The loading of oxide in CCL-C0, CCL-I35, and CCL-I50 was found to be 1.29, 1.30, and 1.21 mg_oxide_ cm^–2^, respectively. All additives were adjusted according to perovskite weight; herein wt % therefore simply refers to the weight percentage of the additive with respect to LCFCO. The general experimental procedure for the electrode method is shown in Figure 1a, and a respective schematic of the CCL composition is shown in Figure 1b.

PtIr(40 wt %)/C(60 wt %) powder was prepared using a borohydride reduction process described elsewhere and was employed as the anode electrode.^32^ In brief, PtIr/C electrocatalysts were synthesized with Pt:Ir atomic ratios of 9:1 using H_2_PtCl_6_·6H_2_O (>37.5%, Sigma-Aldrich) and IrCl_3_ (99.8%, Sigma-Aldrich) as metal sources. The metal sources were added to a mixture of CB dispersed in a propanol/water solution (50/50 v/v) and sonicated for 5 min. The mixture was subsequently stirred, and a solution of NaBH_4_ in 0.1 M KOH was slowly added at room temperature. After thoroughly stirring, the final mixture was filtered and the remaining solid was washed with water and then dried at 70 °C for 2 h. The as prepared powder was labeled PtIr(40 wt %)/C(60 wt %). Utilizing propanol and water as the solvent, the PiperION BP-100 ionomer (20 wt %) was ultrasonicated in an ice–water bath for 1 h with PtIr/C to form a uniform dispersion. The ink was then brushed onto the pretreated carbon cloth, and a loading of 2.2 mg_PGM_·cm^–2^ was obtained.

### 2.4 Physicochemical Characterization

X-ray powder diffraction (XRD) analysis was used to examine the phase and purity of the perovskite powder and catalyst layers. Measurements were carried out at room temperature on a PANalytical X’Pert Pro diffractometer (Cu Kα source, 1.5405 Å) and collected in the 2θ range of 20–80° with a step of 0.0167°. Phase analysis and identification were conducted via the HighScore software.

To evaluate the distribution of grain sizes and account for the noncoherent domains, scanning electron microscopy (SEM) images were taken. SEM was used to examine the morphology of the catalyst layers at the surface using a Zeiss SUPRA 55-VP, equipped with an energy-dispersive X-ray (EDS) spectrometer for elemental and point analysis.

The contact angle of water droplet formation on gas diffusion layer surface with different PTFE loadings was determined by the pictures taken by a smart phone with a macrolens attachment (KINGMAS Macro Clip camera lens). A tangent was then drawn from the points on the droplet that were simultaneously in contact with the solid diffusion layer, liquid, and air. The contact angle was measured using the ImageJ software.

### 2.5 Single-Cell Evaluation Test

The test conditions for the single cell with an active area of 1 cm^2^ was anode fuel, 2 mL min^–1^ 7 M NH_3_H_2_O + 1 M KOH at 3 bar, and cathode fuel, 180 mLmin^–1^ CO_2_-free air fed through a humidifier at a temperature of 95 °C at 2 bar. The cell temperature was held at 80 and 100 °C and the corresponding polarization curves were obtained using a Solartron 1287A Electrochemical Station. Electrochemical impedance spectroscopy (EIS) was conducted on a Solartron 1260A at a frequency range of 1 MHz to 0.01 Hz and fixed potential of 10 mV bias, and the voltage was set to the open circuit potential for each test conducted.

## 3 Results and Discussion

**Figure 2:**
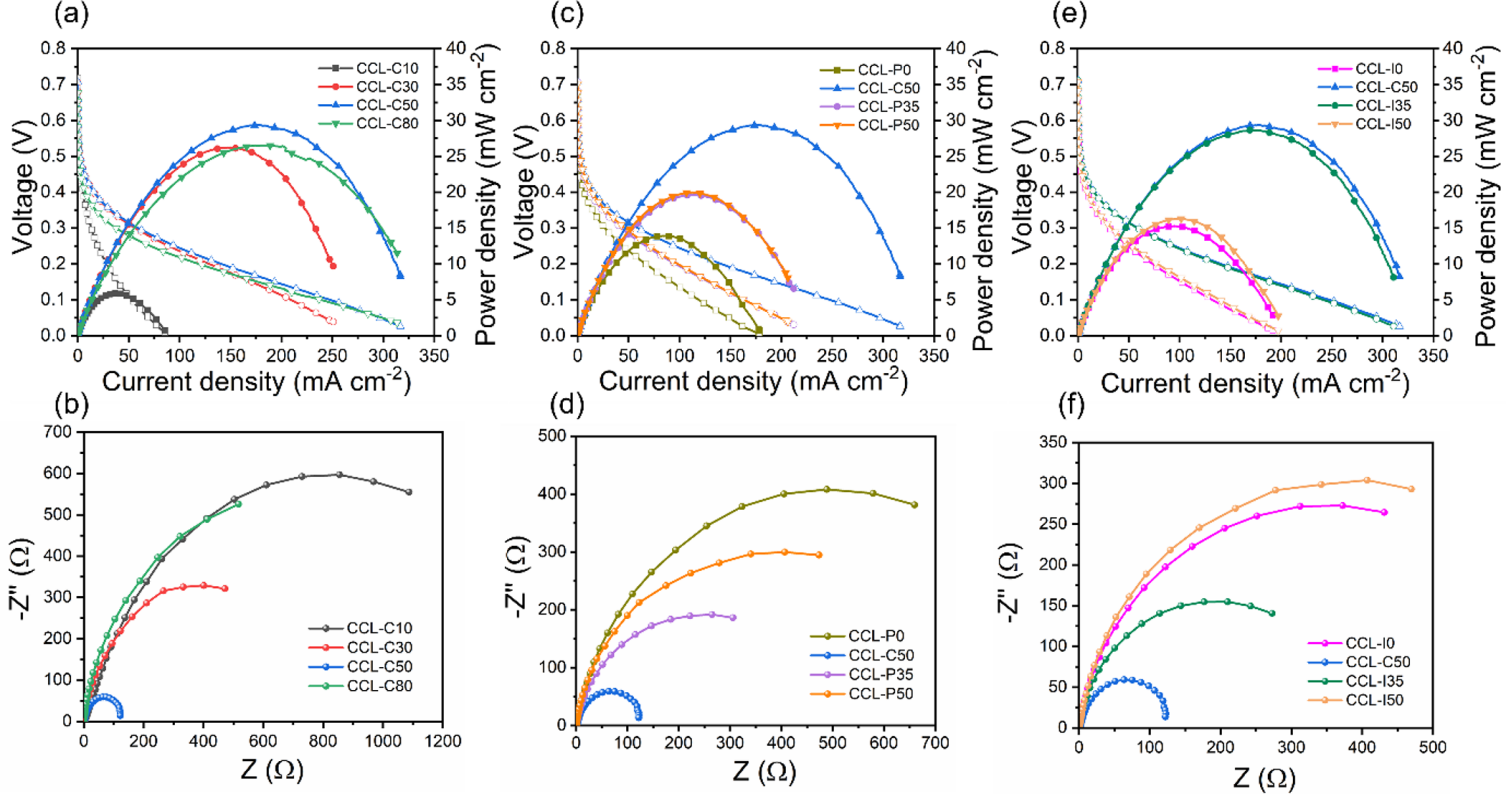
(a) Polarization and power density curve and (b) EIS data of CCLs with varying carbon content. (c) Polarization and power density curve and (d) EIS data of CCLs with varying PTFE content. (e) Polarization and power density curve and (f) EIS data of CCLs with varying ionomer content. Anode: 2.2 mg_PGM_ cm^–2^ PtIr(40 wt %)/C(60 wt %). Cathode: LCFCO. Test conditions: (1) anode, 2 mL min^–1^ of 7 M NH_3_H_2_O with 1 M KOH; anode back pressure, 3 bar_g_; (2) Cathode, 180 mL min^–1^ CO_2_-free air through humidifier of *T* = 95 °C; cathode back pressure, 2 bar_g_. Cell temperature: 80 °C.

The effects of CB, ionomer, and PTFE content on fuel cell performance were sequentially tested. First, a series of CCLs were fabricated based on varying CB amount to find the optimum carbon content. Four DAFCs were assembled where CB in the CCL was varied to 10, 30, 50, or 80 wt %, hereby referred to as CCL-C10, CCL-C30, CCL-C50, and CCL-C80, respectively. Meanwhile, arbitrary amounts of PTFE (10 wt %) and ionomer (20 wt %) were chosen. The CB content delivering optimum performance was then used as a baseline as PTFE content was explored. Three further DAFCs were assembled where PTFE in the CCL was varied to 0, 35, or 50 wt %, hereby referred to as CCL-P0, CCL-P35, and CCL-P50, respectively. As a comparison, CCL-C50 (10 wt % PTFE) was also compared in this series. Upon optimization of both CB and PTFE, ionomer content was investigated. Three further DAFCs were assembled where ionomer in the CCL was varied to 0, 35, or 50 wt %, hereby referred to as CCL-I0, CCL-I35, and CCL-I50, respectively. Again, for comparative purposes, CCL-C50 (20 wt % ionomer) was also compared in this series. Results of the 10 corresponding DAFCs along with their respective EIS data are shown in Figure 2.

Figure 2a–f demonstrate the polarization curves and EIS data of the different CCLs employed within a DAFC operating at 80 °C, the results of which are highlighted in Table 1.

**Table 1:** DAFC Performances Based on Varying Carbon, PTFE and Ionomer Content in CCLs^a^

optimization		carbon (wt %)	PTFE (wt %)	ionomer (wt %)	OCV (V)	maximum current density (mA cm^–2^)	PPD (mW cm^–2^)
carbon	CCL-C10	10	10	20	0.72	85.3	5.92
	CCL-C30	30	10	20	0.71	250	26.2
	CCL-C50	50	10	20	0.71	317	30.1
	CCL-C80	80	10	20	0.70	312	26.5
PTFE	CCL-P0	50	0	20	0.69	179	13.9
	CCL-P35	50	35	20	0.71	206	20.1
	CCL-P50	50	50	20	0.71	212	19.7
ionomer	CCL-I0	50	10	0	0.65	192	15.2
	CCL-I35	50	10	35	0.71	311	28.6
	CCL-I50	50	10	50	0.71	197	16.3

a All wt % given with respect to perovskite weight.

The individual influence of varying CB, PTFE, and ionomer content was investigated in greater detail and is discussed in the following sections. To understand the effects of each component added, it is useful to consider the fuel cell performance in three regimes: (i) activation polarization losses, (ii) ohmic polarization losses, and (iii) concentration polarization losses which affect the low, middle, and high current density regimes, respectively.^1,33^

### 3.1 Effect of Carbon Black Content

The polarization and EIS data of the DAFCs with varying CB amount in the CCL are shown in Figure 2a,b.

Open circuit voltage (OCV), current density, and peak power density (PPD) are often quoted in the literature and are used as parameters to judge fuel cell performance. The OCV values remained fairly unchanged among the four cells, with values of 0.72, 0.71, 0.71, and 0.70 V being obtained for CCL-C10, CCL-C30, CCL-C50, and CCL-C80, respectively. The results indicate that the effect of carbon content on OCV is minimal. The current density shows a substantial improvement as the CB content is varied from 10 to 30 wt %, with values of 85.3 and 250 mA cm^–2^, respectively. As CB content is increased to 50 wt %, this further increases to 317 mA cm^–2^. In CCL-C80 however, there is a slight decrease in current density to 312 mA cm^–2^. More notably perhaps is that the PPD shows a great improvement, from 5.92 to 30.1 mW cm^–2^ as CB content is increased from 10 to 50 wt %. This demonstrates a performance increase of nearly 5 times by properly tailoring carbon content, highlighting the crucial significance and need to explore carbon ratios within the CCL.

When examining the polarization curves in greater detail, further information regarding the effects of carbon content within the CCL can be extracted. The DAFC employing CCL-C10 shows a substantial drop in voltage at lower current density compared to the cells with higher carbon content. The overpotential associated with this regime arises from activation losses. It is known that this activation overpotential (η_a_) is linked to electron transfer at the electrode–electrolyte interface. Electron accumulation, for example, due to inefficient electron transport at the electrode surface, may produce an energy barrier for incoming electrons.^34^ Since perovskite oxides have inherently low electrical conductivity, increasing the content of carbon in the catalyst layer can assist in reducing this overpotential by creating an electronic network. This is evidenced in the DAFCs employing CCL-C30, CCL-C50, and CCL-C80 which exhibit a smaller drop in voltage at lower current densities.

Furthermore, carbon black in CCLs can influence the extent of ohmic losses since these losses occur partially due to the resistance of electron flow through electrically conductive fuel cell components.^35^ Thepkaew et al., for example, found that ohmic losses are heavily influenced by carbon.^36^ Referring to the middle regime of the polarization curves which is closely linked to ohmic overpotential (η_o_), the drop in voltage becomes less prevalent as carbon content is increased from 10 to 50 wt %. The results imply that the extent of ohmic losses is reduced upon increasing carbon amount throughout the CL. Since the remaining cell components such as electrolyte, gas diffusion layers (GDLs), flow field plates (FFPs), current collectors, and interface contacts are unchanged throughout the tests, this can be owed to the superb electronic conductivity of carbon which provides a sufficient network for electrons to travel to and from active sites of the catalyst layer. As carbon content is further increased to 80 wt %, there is a slight decrease in performance. Nonetheless, the overall improvement in performance as carbon content is increased reinforces the notion that carbon black can facilitate electrical contact between the oxide particles and help eliminate issues relating to electronic conductivity.^37^

To study resistance of the cells, EIS of the cells was carried out. EIS data are commonly fitted to an equivalent circuit which combines resistances and specific electrochemical elements such as the double layer (modeled as capacitance) as well as inductance and Warburg diffusion elements.^38,39^ A constant phase element (CPE) tends to substitute the capacitance in electrochemical circuits due to the inhomogeneity of the testing conditions such as electrode roughness, coating, and distribution of reaction rate.^38^ A simple equivalent circuit for electrochemical devices such as the Randles circuit include the combined effects of solution resistance (*R*_s_), anodic polarization resistance (*R*_a_), and cathodic polarization resistance (*R*_c_). The sum of such resistances (*R*_s_, *R*_a_, and *R*_c_) can be modeled as *R*_in_. In systems where ohmic resistance is strong, such as in fuel cells, EIS can be employed to efficiently measure *R*_in_, which takes into consideration resistances at the electrode–electrolyte interface.^38,39^

The plot in Figure 2b shows EIS data of the different DAFCs employing CCLs with varying carbon content. The resistance includes the combined effects mentioned above and can be modeled as *R*_in_. Since the remaining components within the fuel cell are constant throughout the different tests, the change in EIS data can be owed to the varying CCLs employed and therefore more closely resemble the cathode activation loses. As carbon content is increased from 10 to 50 wt %, there is an obvious improvement in resistance which is evidenced by smaller semicircles. The results imply that there may be better contact at the electrode–electrolyte interface. This can be owed to the large surface and contact area provided by Vulcan XC-72R. Since bulk perovskite oxides tend to be highly sintered due to the elevated temperatures required for phase formation, these materials tend to have a low specific surface area (of order of 2–10 m^2^ g^–1^). This leads to poor mass activity toward oxygen electrocatalysis.^40,41^ Vulcan XC-72R, on the other hand, has a specific surface area of 223 m^2^ g^–1^ and is often used as a support to widen the surface utilization of perovskites.^20,37,42^Table 2 lists surface area values for LCFCO-700 and Vulcan XC-72R; Pt/C is also included for comparison. The BET surface area for commercial Pt/C is provided in the specification sheet from Alfa Aesar, where the Pt/C was originally purchased. Ultimately, as carbon content is increased, resistance is reduced and there is better utilization of the perovskite catalyst. Moreover, when the carbon content was excessive and increased to 80 wt %, there was an increased resistance present within the electrode. It is reasonable to assume that this may be due to partial blockage of the catalyst, making it difficult for O_2_ to reach the active sites for the reaction to occur.^43^ Henceforth an excessive amount of carbon present within the CCL may begin to limit adequate contact of O_2_ with catalyst particles, thus increasing the electrode polarization resistance and in turn limiting cell performance.

**Table 2:** Surface Area of Materials Used in Catalyst Layers

material	BET surface area (m^2^ g^–1^)
Pt/C	90
LCFCO	5.2^24^
Vulcan XC-72R	223^42^

The need to optimize carbon content in CCLs is clear and is in accordance with literature where carbon is extensively used as a conductive support for a wide range of electrocatalysis applications due to its good electrical conductivity, low cost, good chemical stability, and high surface area.^1,20^ In this study, an optimum carbon black weight of 50 wt % with respect to the perovskite oxide was found to provide optimum DAFC performance and a sufficient electron network throughout the entire gas diffusion electrode (GDE).

### 3.2 Effect of PTFE Content

Since there is obvious evidence of flooding at the cathode due to liquid crossover, as reported by Wang et al., water management is still of interest in CCLs in DAFCs.^26^ Study into the effects of PTFE content and hydrophobicity of the CL is consequently imperative. The polarization and EIS data of the DAFCs with varying PTFE amount in the CCL are shown in Figure 2c,d.

OCV of the four cells showed minimal deviation, with values of 0.69, 0.71, 0.71, and 0.71 V being obtained for CCL-P0, CCL-C50, CCL-P35, and CCL-P50, respectively. The results imply that the presence of varying PTFE content had minimal impact on OCV. In the absence of PTFE, the DAFC shows poorest performance, with a maximum current density and PPD of 179 mA cm^–2^ and 13.9 mW cm^–2^, respectively. As PTFE content is increased to 10 wt %, performance almost doubles, with a maximum current density and PPD of 317 mA cm^–2^ and 30.1 mW cm^–2^, respectively. It is well recognized that binders such as PTFE often form a porous CL that efficiently facilitates hydrophobic channeling and transport of reactants and products.^2,4−6,25,29,44−48^ This is due to the creation of void regions, which may otherwise be flooded by water, and can provide mass transport channels to and from the active site of the catalyst. On further increase of PTFE content, however, performance declines, with PPDs of 20.1 and 19.7 mW cm^–2^ in CCL-P35 and CCL-P50, respectively.

When more carefully examining the polarization curves, the performances of the cells vary greatly at the middle and high current density regime. In the DAFC that employs CCL-P0 which has no PTFE, there is a large voltage drop within these regions. This may be due to water presence filling void regions within the CCL and blocking potential pathways for the supply of fresh fuel and oxidant as well as the route for product removal. This subsequently leads to losses from concentration overpotential (η_c_) and may explain the low voltage at high current density. Furthermore, this implies a lack of catalyst utilization within the CL and potential hindrance to the access of carbon particles which leads to electronic resistances that may explain the low voltage at medium current density.^46^ The DAFC employing CCL-C50 which contains 10 wt % PTFE showed a great improvement in performance and concentration overpotential. As PTFE content is further increased, however, the DAFCs employing CCL-P35 and CCL-P50 show a similar drop in voltage at medium and high current density, again owed to ohmic and concentration overpotentials, respectively. This drop in voltage may be predominantly owed to blockage of the gas passageways by excessive PTFE and incomplete utilization of the catalyst. Similar findings have been reported in literature, where PTFE content is often kept relatively low to avoid such issues.^46^

**Figure 3:**
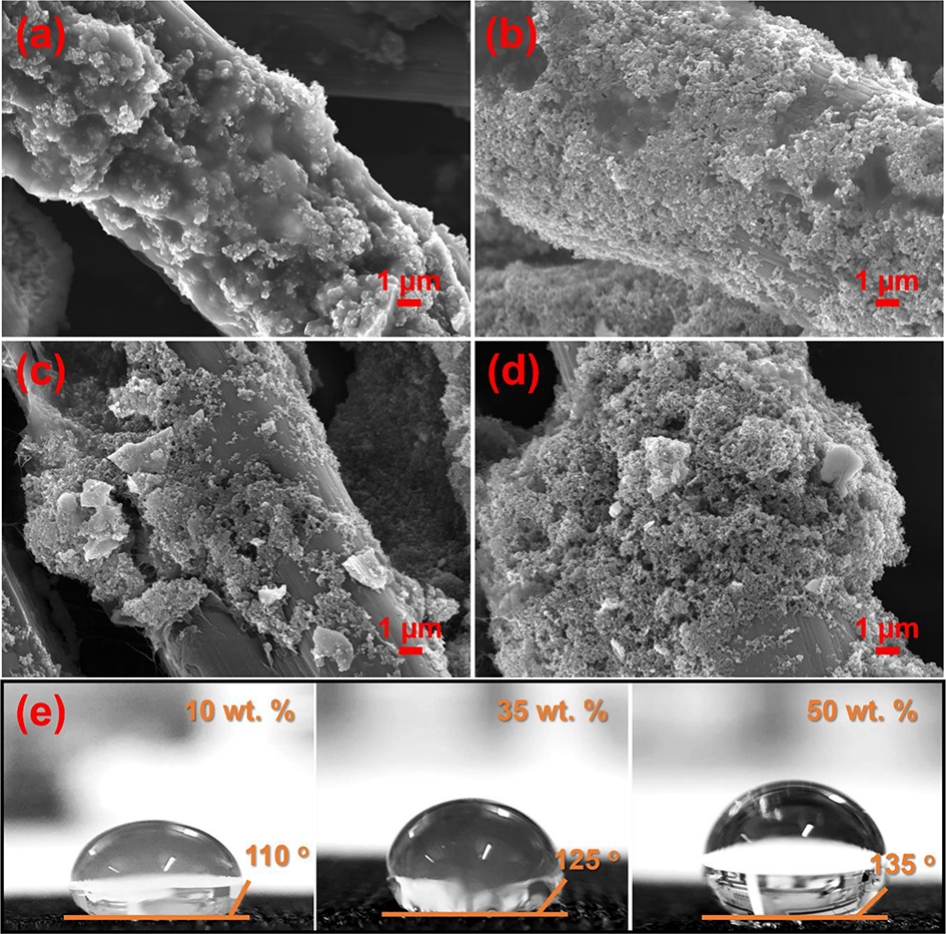
SEM images of (a) CCL-P0, (b) CCL-C50, (c) CCL-P35, and (d) CCL-P50. (e) Water droplet formation on gas diffusion layer surface with different PTFE loadings reflective of 10, 35, and 50 wt % with respect to perovskite.

Since the catalyst ink consists of multiple elements such as the perovskite oxide powder, carbon black, PTFE, and PiperION BP-100 ionomer in a solvent consisting of alcohol and water, there are various contributions to shape, size, and surface properties making interactions fairly complex to analyze independently.^44^ The effect of PTFE content was subsequently visualized via morphological characterization through SEM (Figure 3a–d). The gray color reflects the CCLs deposited onto the carbon cloth diffusion layers. In CCL-C50, the CL remains fairly homogeneous. Catalyst agglomeration, however, was clearly observed at higher PTFE concentrations, in both CCL-P35 and CCL-P50. The presence of agglomeration upon increased PTFE content is expected and has been reported previously in literature.^49^ Such agglomeration can lead to reduced catalyst utilization and blockage of pathways, leading to disruption of electronic and ionic networks and a loss in the electrochemical surface-active area. Furthermore, the presence of agglomeration can lead to uneven CL surfaces and thicker electrodes which leads to higher diffusion resistances, reactant starvation, and localized dehydrated or flooded regions in the catalyst layer.^25^ These results may explain the decrease in DAFC performance with higher PTFE content in the CCL. To validate the phenomenon of large water coverage in the absence of PTFE, carbon cloths were prepared with the same PTFE loadings as in the CCLs and a water droplet was deposited to explore interactions at the GDL surface. The contact angle was measured using the ImageJ software, and results are shown in Figure 3e. It should be noted that only the samples containing PTFE are shown here since the sample containing no PTFE was difficult to capture due to quick absorption into the carbon cloth layer. Nevertheless, the overall trend can clearly be identified among the three samples tested, with an increase in PTFE unequivocally increasing the contact angle of water on the surface, similar to that reported in literature.^26^ Namely, the contact angle increases from 110 to 135° as the PTFE wt % is increased from 10 to 50. This indicates that addition of PTFE is likely to reduce risk of water flooding. However, it should be noted that although increasing PTFE content is ideal with respect to water flooding, it can cause blockage by agglomeration as shown by SEM. The two imaging techniques combined suggest that enough PTFE must be added to avoid water flooding and to create void regions for hydrophobic channelling but not excessively such that there is blockage of gas to and from the active sites due to agglomeration.

To explore the combined resistance throughout the cells, EIS data were analyzed and results are shown in Figure 2d. In the absence of PTFE, there is a large resistance most likely due to high water amount in the CCL causing flooding at the cathode, making it difficult for gas to diffuse through the layers and reach the active sites.^25^ Increasing PTFE content was paralleled by a notable reduction in the size of the semicircle in CCL-C50, implying improved mass transfer. It has been noted that an increase in porosity of the electrode is known to play a key role in diminishing mass transfer resistance by lowering saturation in the cathode GDL by introducing hydrophobic channeling among pores and avoiding flooding at the electrode.^25,46,47^ As PTFE content is increased further, however, there is an increase in resistance, indicated by the larger arc sizes for CCL-P35 and CCL-P50. These results correlate well with the polarization curves and may be owed to blockages of the active catalyst on the electrode surface causing limitations for the proceeding reaction.

The results demonstrate that an optimum PTFE content is essential in providing hydrophobic channeling for efficient water management while limiting the formation of agglomerates. A trade-off is therefore crucial and should be considered as a part of the CCL design. In this study, an optimum PTFE weight of 10 wt % with respect to the perovskite oxide was found to provide optimum DAFC performance and provide sufficient porous network throughout the entire GDE.

### 3.3 Effect of Ionomer Content

**Figure 4:**
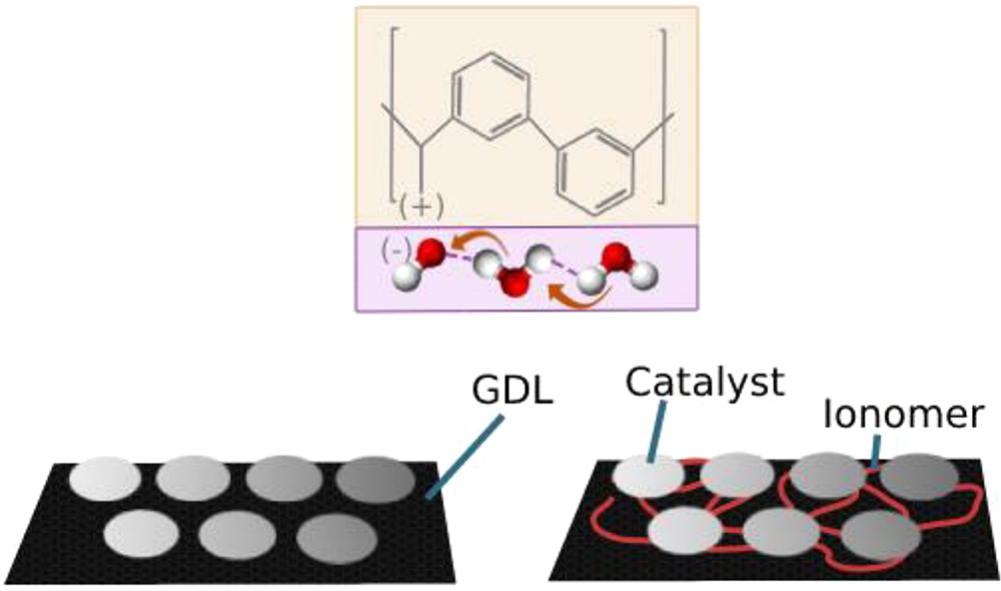
Proposed OH^–^ mechanism through the hydrogen-bonded water network solvated by hydrophilic side chains (top) and illustration of an efficient ionic network on introduction of ionomer in the CL (bottom).

The ionomer is designed to be physically compatible with the membrane employed and possess high ionic conductivity, negligible electronic conductivity, and high gas/water permeability.^1^ This study utilizes a polymer based PiperION BP-100 ionomer that has recently been documented to exhibit excellent hydroxide conductivity.^30^ Simulation studies have indicated that ionic current flow may occur via an OH^–^ hopping mechanism through the hydrogen-bonded water network solvated by hydrophilic side chains of the polymer, as shown in Figure 4.^1,4,50−56^ To benefit from increased ionic conductivity throughout the CL, a good network and connectivity for the OH^–^ hoping mechanism must therefore be established.^50^ This study exploits a PiperION BP-100 ionomer alongside a PiperION-A20-HCO3 TP-85 membrane to ensure a compatible interface and to create an effective network for hydroxide transportation to take place across the MEA to the active sites of the CL. Exploration into the ionomer content to manipulate such networks is therefore crucial for good DAFC performance. The polarization and EIS data of the corresponding DAFCs with varying ionomer amount in the CCLs are shown in Figure 2e,f.

The DAFC employing a CCL with no ionomer shows a lower OCV of 0.65 V compared to its counterparts. As ionomer content is increased in CCL-C50, CCL-I35, and CCL-I50, the OCV values increase, with 0.71 V being obtained for all three cells. Since the reactions that take place at the anode and cathode sites of a DAFC involve hydroxide ions, this is most likely owed to the lack of an effective ionomer network to mediate the transfer of hydroxide ions to the catalytic active sites for the given reactions to occur. The maximum current density and PPD show great improvement on addition of ionomer, with values of 192 and 317 mA cm^–2^ and 15.2 and 30.1 mW cm^–2^ being achieved for DAFCs employing CCLs with 0 and 20 wt % ionomer, respectively. Notably, the PPD is nearly doubled when ionomer content increased to 20 wt %, highlighting the importance of ionomer addition. As ionomer content is further increased, the performance gradually drops with PPDs of 28.6 and 16.3 mW cm^–2^ obtained when CCLs with 35 and 50 wt % were implemented, respectively.

The large ohmic loss present in the DAFC employing CCL-I0 indicates an insufficient ionic network causing a large ionic resistance and a decrease in performance due to poor ionic transfer between the electrolyte and catalyst. On addition of ionomer, the DAFC employing CCL-C50, which has 20 wt % ionomer, shows better performance due to capability of forming a well-connected three-phase boundary.^45^ This is expected since good ionomer distribution in CLs is known to increase connectivity to the number of active sites. This increases the electrochemical surface area and forms a more continuous ionic transport network channel, enhancing ORR activity at the cathode site.^5,57^ As ionomer content is further increased in CCL-I35 and CCL-I50, the performance begins to decrease and there is notable reduction in voltage within the medium and high current density regimes. Although addition of ionomer is required for good ionic conduction, it is well-known that excessive ionomer coverage blocks active sites on the catalyst.^6,58−60^ Oxygen diffusion paths to the active sites therefore become harder, which significantly increases mass transport resistance and slows down the rate of ORR.^5,6,58−60^ Similar phenomena are observed in literature and indicate that a trade-off in the electrode must be made.^5,6,45,58−60^ Furthermore, it is possible for the ionomer network to block electron conductivity due to coverage of the carbon surface, leading to a decrease in complete utilization of the CCL surface.^47^ This may also contribute to the increase in ohmic resistance observed, specifically electron resistance, and may explain the ohmic overpotential experienced at relatively high ionomer content.

To further explore resistance, EIS data were analyzed and are shown in Figure 2f. As ionomer content increases, resistance also increases. This is not uncommon as ionomers are known to partially cover the catalyst active sites and reduce reactant permeability.^45,61,62^ Addition of ionomer content into the CCL will therefore unequivocally enhance mass transport resistance. This effect is amplified as ionomer content is increased, with CCL-I50 displaying the largest semicircle at low frequency. Enough ionomer must therefore be added to form a well-connected triple-phase boundary (TPB) but not to completely block access to catalyst active sites, which can increase mass transfer resistance.

**Figure 5:**
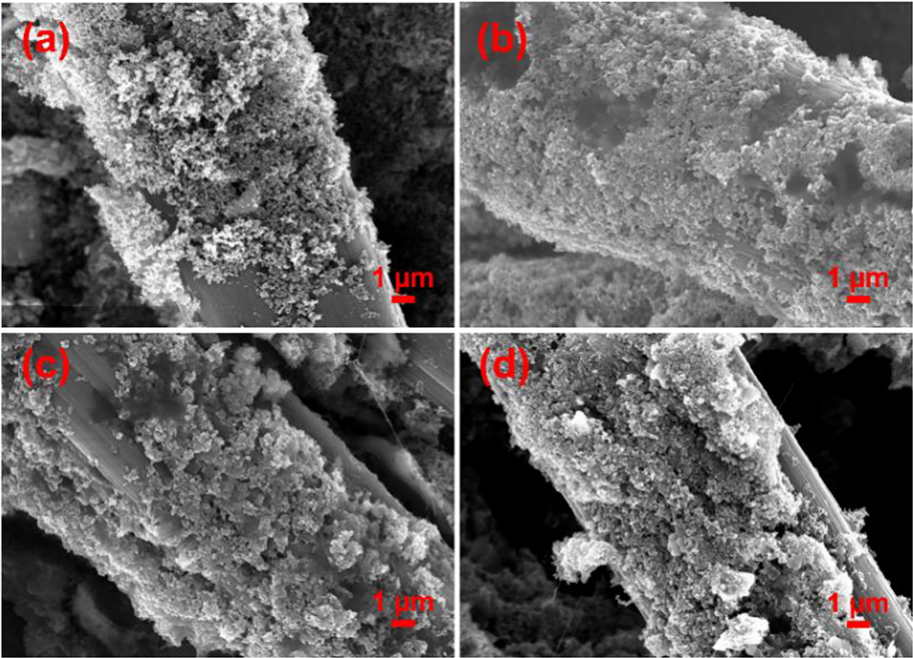
SEM images of (a) CCL-I0, (b) CCL-C50, (c) CCL-I35, and (d) CCL-I50.

To investigate surface morphology, SEM was conducted and is shown in Figure 5. It should be recognized that when ionomer content within the CCL was 20 wt %, particles are more uniformly distributed. This may explain the enhanced mass transport and adequate formation of an ionic conducting network compared to higher ionomer content.^63^ In CCL-I35 and CCL-I50, there is no longer homogeneous coverage, which may indicate partial blockage of mesopores and additional resistance of oxygen transport through the CCL.^48,64,65^ As ionomer content is increased to 50 wt % in particular, agglomerates can clearly be observed within the CCL. Agglomeration has been widely studied when Nafion is used as an ionomer.^1,4,44,66^ Three effects have been of significant interest: (i) the effect of ionomer on self-agglomeration, (ii) its ability to form a coating layer on carbon black, and (iii) the effect of ionomer on polymer interaction between carbon black aggregates.^4^ Formation of ionomer agglomerates is therefore common and a similar effect may subsequently be present with the PiperION BP-100 ionomer, explaining the increased mass transfer resistance and lowered DAFC performance at higher ionomer content.^44,66^ Moreover, it is possible for an ionomer network to block electron conductivity due to coverage of the carbon surface, leading to a decrease in catalyst utilization.^47^ This may also contribute to the increase in ohmic resistance at particularly high ionomer content since ohmic losses are affected by both ionic and electronic conductivity.

The above results imply that an optimum ionomer distribution is needed and a compromise must be considered in the design of CCLs.^2,4−6,25,29,44−48,58−60,65^ In this study, an optimum ionomer weight of 20 wt % with respect to the perovskite oxide was found to provide optimum DAFC performance and a sufficient ionomer network throughout the entire GDE. This 4:1 ratio of catalyst:ionomer is widely used in literature and therefore the data in this study agree with these findings.^30,67^

### 3.4 Varying Operating Conditions

To truly appreciate the scope and applicability of CCLs, the DAFCs must be tested under varying conditions. The following section therefore explored the CCLs under different operational conditions such as temperature and hydroxide concentration.

**Figure 6:**
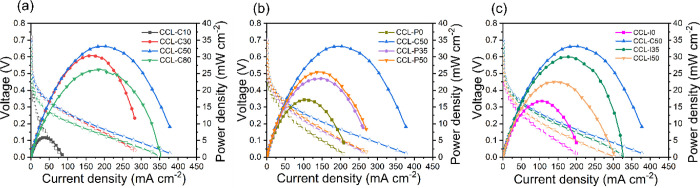
(a) Polarization and power density curve of CCLs with varying (a) carbon, (b) PTFE, and (c) ionomer content. Anode: 2.2 mg_PGM_ cm^–2^ PtIr(40 wt %)/C(60 wt %). Cathode: LCFCO. Test conditions: (1) anode, 2 mL min^–1^ of 7 M NH_3_H_2_O with 1 M KOH; anode back pressure, 3 bar_g_; (2) cathode, 180 mL min^–1^ CO_2_-free air through humidifier of *T* = 95 °C. Cathode back pressure: 2 bar_g_. Cell temperature: 100 °C.

The cells were subsequently also operated at 100 °C as displayed in Figure 6 to explore the effects of temperature. The overall trends observed among the DAFCs with adjusted carbon, PTFE, and ionomer content at this operational temperature followed the same trend as was seen in sections 3.1–3.3 where the operational temperature was 80 °C, with CCL-C50 showing optimum performance among the series of CCLs tested. As expected, the performance increases with an increase in temperature and the DAFC employing CCL-C50 again exhibits optimum performance, with a maximum current density of 379 mA cm^–2^ and PPD of 34 mW cm^–2^. This positive increase in DAFC performance with an increase in operating temperature has commonly been observed in literature.^31,68^ More importantly, it demonstrates that the cell can be held at elevated temperatures without loss of functionality.

**Figure 7:**
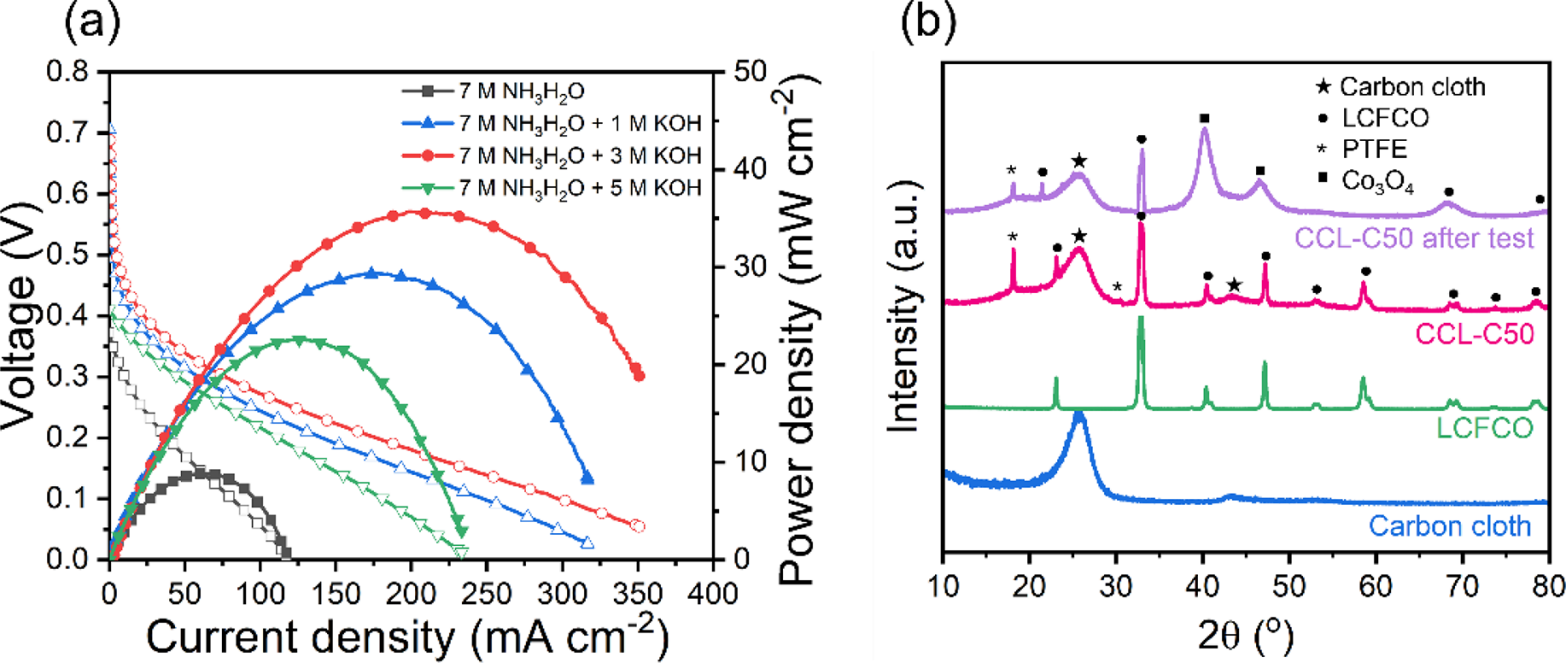
(a) Polarization and power density curves of DAFC employing CCL-C50 fed with 7 M NH NH_3_H_2_O and different hydroxide concentrations. Anode: 2.2 mg_PGM_ cm^–2^ PtIr(40 wt %)/C(60 wt %). Cathode: 1.23 mg_oxide_ cm^–2^ LCFCO. Test conditions: (1) anode, 2 mL min^–1^ of 7 M with 1 M KOH; anode back pressure, 3 bar_g_; (2) Cathode, 180 mL min^–1^ CO_2_-free air through humidifier of *T* = 95 °C. Cathode back pressure: 2 bar_g_. Cell temperature: 80 °C. (b) XRD of carbon cloth, LCFCO perovskite oxide, CCL-C50 before and after fuel cell durability test.

Since CCL-C50 was identified as the optimized composition, this catalyst layer was employed as the cathode for the following tests. The effects of hydroxide concentration in the electrolyte were also studied and are shown in Figure 7a. These tests were conducted at an operating temperature of 80 °C. In the absence of hydroxide, the cell was still able to produce a maximum current density and PPD of 118 mA cm^–2^ and 8.8 mW cm^–2^, respectively. This is particularly useful if considering DAFCs for wastewater treatment technology since the high ammonia content in wastewater can be utilized to power such cells without the need for hydroxide addition, eliminating postpurification processing and additional energy input.^8^ It should be noted, however, that a lower OCV of 0.38 V was obtained in the lack of KOH, most probably since the reaction mechanisms involve OH^–^ ions and absence of such affects the reactions at the respective electrodes which are shown below (eqs 1–3).^14^
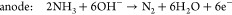
1
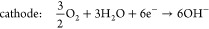
2
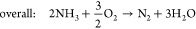
3

As hydroxide concentration in the electrolyte is increased to 3 M, there is an increase in performance, with an OCV, maximum current density, and PPD of 0.71 V, 351 mA cm^–2^, and 35.6 mW cm^–2^ being achieved, respectively. The increase in performance is likely due to greater availability of hydroxide ions to assist the electrode reactions. As hydroxide concentration is further increased to 5 M, however, the performance begins to decline. A similar trend in DAFC performance as hydroxide performance is excessively increased has also been observed in literature and is presumed to be due to transport limitations.^9^

It is interesting to note that in our previous study, we used a similar setup and employed a Pt/C-based CCL. This DAFC showed an OCV and PPD of 0.60 V and 32 mW cm^–2^ under conditions of 7 M NH_3_ + 1 M KOH as the anode stream, humidified air as the cathode stream, and a cell temperature of 80 °C.^24^ The results of this study show not only that under similar operating conditions can a superior OCV (0.71 V) and similar PPD (30.1 mW cm^–2^) be attained when using a non-PGM-based CCL but that by simply changing the composition of the fuel (i.e., 3 M KOH), CCL-C50 can outperform the Pt/C-based CCL.

Figure 7b reveals XRD data of the CCL-C50 electrode before and after testing. Compared to the pure perovskite oxide powder, additional peaks at 2θ values of 18.0° and 31.5° arose due to the (100) and (110) planes of PTFE (PDF: 00-060-1504) in CCL-C50 as expected. XRD analysis also reveals the presence of the carbon cloth substrate which is included for reference. After testing, however, there are obvious losses in LCFCO-700 peaks, implying loss of active sites which may hinder performance after long testing durations. Moreover, the presence of reflections at 2θ values of 40.6 and 47.3° belonging to the (222) and (400) planes of Co_3_O_4_ (PDF: 04-022-7367), respectively, indicates that there may be structural changes after testing.

**Figure 8:**
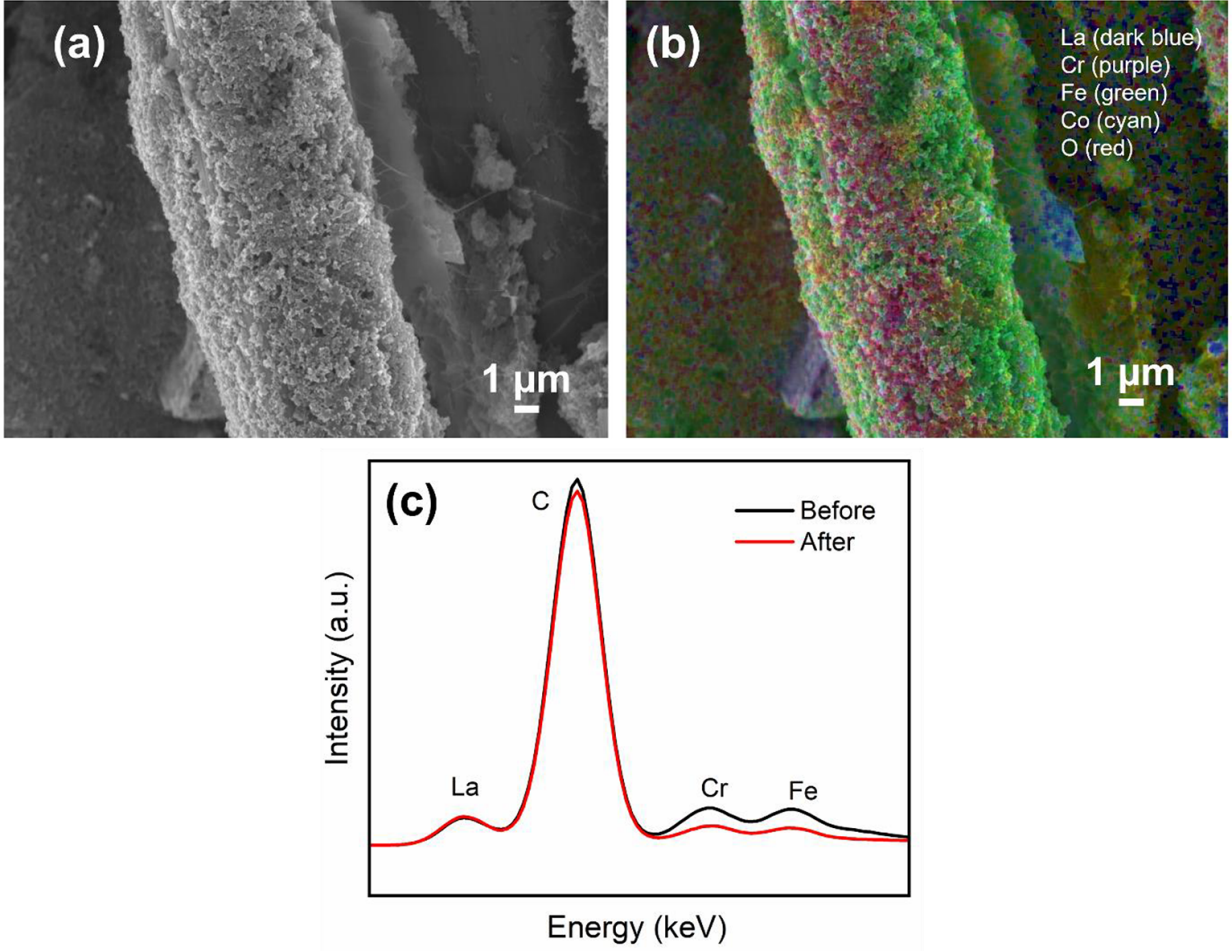
(a) SEM of CCL-C50 after testing. (b) Elemental mapping of CCL-C50 with overlap of elements La (dark blue), Cr (purple), Fe (green), Co (cyan), and O (red). (c) Point analysis of CCL-C50 before and after durability test.

The SEM and EDS images presented in Figure 8a,b show that the La, Cr, Fe, Co, and O elements remain homogeneously distributed. The point analysis data in Figure 8c show that the relative intensities of the La and F elements which arise from the LCFCO and PTFE components of the CCL, respectively, decrease after testing. This further reinforces loss of active sites and prompts room for improvement in the durability of CCL design.

## 4 Conclusion

To maximize performance of the membrane electrode assembly (MEA), transport pathways for electrons, ions, and reactants should be connected well. This demands a well-constructed microstructure in the catalyst layer (CL). It is widely known such a CL is a multicomposite structure composed of catalysts, carbon supports, ionomer materials, and void regions, whereby carbon and ionomers are used to provide electron and ionic conduction networks, respectively, and void regions provide mass transport channels for reactants and products. An effective three-phase interface design is therefore necessary for improving cell performance and in reducing CL costs. Herein we design and optimize a cathode CL (CCL) for a direct ammonia fuel cell (DAFC) using a perovskite oxide as the catalyst to reduce reliance on platinum group metals (PGM). The effects of tailoring carbon, ionomer, and PTFE content in the CCL were explored, and several DAFCs were tested. Using the same catalyst and operating conditions, the lowest maximum current density and peak power density obtained were 85.3 mA cm^–2^ and 5.92 mW cm^–2^, respectively, which substantially increased to 317 mA cm^–2^ and 30.1 mW cm^–2^ through proper carbon, ionomer, and PTFE optimization, illustrating the importance of an effective three-phase interface. The findings reveal that despite employment of an active catalyst for oxygen reduction at the cathode site, the true performance of the catalyst cannot be reflected unless it is supported by proper design of the CCL. The study also reveals that by optimizing the CCL, similar performances to those of Pt/C based CCLs stated in literature can be attained. Given that the cost reduction is substantial and the performance is fairly similar, we believe that the design and optimization of non-PGM based CCLs is a crucial route for fuel cell progression.
